# Clinical and genomic characteristics of mucosal signet-ring cell carcinoma in *Helicobacter pylori*-uninfected stomach

**DOI:** 10.1186/s12876-020-01387-9

**Published:** 2020-07-29

**Authors:** Mariko Kiso, Yuji Urabe, Masanori Ito, Kazuhiko Masuda, Tomoyuki Boda, Takahiro Kotachi, Kosaku Hata, Naoki Yorita, Naoko Nagasaki, Madina Abduwali, Yuich Hiyama, Shiro Oka, Shinji Tanaka, Kazuaki Chayama

**Affiliations:** 1 Department of Gastroenterology and Metabolism, Hiroshima University, Hiroshima, Japan; 2 Department of Medicine and Molecular Science, Division of Frontier Medical Science, Programs for Biomedical Research, Graduate School of Biomedical Sciences, Hiroshima University, Hiroshima, Japan; 3 Department of General Internal Medicine, Hiroshima University Hospital, Hiroshima, Japan; 4 Department of Gastroenterology, Miyoshi Central Hospital, Hiroshima, Japan; 5 Department of Endoscopy, Hiroshima University Hospital, Hiroshima, Japan; 6 Department of Gastroenterology, Chugoku Rosai Hospital, Hiroshima, Japan

**Keywords:** *Helicobacter pylori*, Uninfected, Signet ring cell carcinoma, Next generation sequence, Smoking

## Abstract

**Background:**

Gastric cancer develops even in *Helicobacter pylori*(*H. pylori*)-uninfected patients and its typical histological feature is signet ring cell carcinoma (SRCC) within the mucosal layer. However, the biological characteristics of SRCC remain unclear. We aimed to clarify the pathological and genetic features of SRCC in *H. pylori*-uninfected patients.

**Methods:**

Seventeen *H. pylori*-uninfected patients with mucosal SRCCs were enrolled and their clinicopathological characteristics were compared with those of *H. pylori*-infected patients with mucosal SRCCs. Seven SRCCs without *H. pylori-*infected, including two invasive SRCCs, and seven *H. pylori*-infected SRCCs were subjected to a genetic analysis using next-generation sequencing.

**Results:**

*H. pylori*-uninfected patients with mucosal SRCCs revealed male dominancy and a significantly higher prevalence of smokers among them as compared with the *H. pylori*-infected patients with SRCC. A *CDH1* mutation (frame shift indel) was detected in one *H. pylori*-uninfected cancer not only in the mucosal SRCC but also in the invasive portion. A *TP53* mutation was detected in one SRCC without *H. pylori-*infected. In the control group, *ARID1A* and *TP53* mutations were detected in one SRCC each. The C to A mutation, which is a characteristic smoking-induced mutation, was not found in any of the samples.

**Conclusions:**

Some SRCCs in *H. pylori*-uninfected patients may have a malignant potential similar to that of SRCCs in *H. pylori*-infected patients. Smoking may not be the main carcinogenic factor for the development of SRCCs among the *H. pylori*-uninfected patients.

## Background

It is well recognized that *Helicobacter pylori* (*H. pylori*)-infected is the main cause of gastric cancer development [1]. In Japan, we previously reported that over 99% of patients with gastric cancer had *H. pylori-*infected [2], and another study reported a similar result [3]. However, gastric cancer may develop even in *H. pylori*-uninfected patients and its typical histological feature is signet ring cell carcinoma (SRCC) within the mucosal layer [4]. Endoscopically, SRCCs can be easily recognized as whitish depressed or flat lesions, and their prevalence and clinical importance have recently been increasing [5].

Previous reports have suggested that SRCCs in *H. pylori*-uninfected patients show a lower proliferative activity, few extensive spread, and slower progression, compared with SRCCs with *H. pylori*-infected patients [6]. The features of SRCCs in *H. pylori*-uninfected patients resemble those in patients with hereditary diffuse gastric cancer (HDGC), which is caused by a germline mutation of *CDH1* [7]. In patients with HDGC, a large number of SRCCs can be detected; however, most of them show little tendency to invade into the submucosal layer. Therefore, it is controversial whether SRCCs in *H. pylori*-uninfected patients can invade into the submucosal layer. In addition, the pathogenesis of SRCCs in *H. pylori*-uninfected patients remains uncertain. Recently, Horiuchi et al. reported the implication of smoking in the disease pathogenesis of these patients, noting that their findings should be verified by other studies [8].

In the present study, we compared the clinical features of SRCCs between patients with and without *H. pylori-*infected. Moreover, we investigated the genomic characteristics of these lesions to identify their biological characteristics and assess whether smoking may be involved in their pathogenesis.

## Methods

### Patients

We enrolled 19 consecutive patients with SRCC (17 with mucosal cancer and 2 with invasive cancer) without *H. pylori*-infected who were diagnosed in Hiroshima University Hospital from 1998 to 2017. All the patients received endoscopic or surgical resection. The entire tumor was cut into parallel 2–4 mm-thick sections for investigation. Histological evaluation was performed according to the Japanese gastric cancer treatment guidelines [9]. Clinicopathological data was retrospectively reviewed from clinical records. The definition of *H. pylori*-uninfected subjects was judged by our criteria as described in a previous publication [2].

A total of 34 patients with *H. pylori*-infected SRCC diagnosed in the same period were enrolled as controls. We compared the clinical characteristics (sex, age, location, size, blood type, family history of gastric cancer, alcohol, smoking, and Brinkman Index) between the two groups (17 patients *H. pylori*-uninfected mucosal SRCC and 34 patients controls). Furthermore, we randomly selected seven patients who were habitual smokers among both the *H. pylori*-uninfected and -infected SRCCs, for next-generation sequencing DNA analysis. This retrospective study was approved by the institutional review board and the ethics committees of Hiroshima University (No. E-1096) and was performed in accordance with the Helsinki Declaration and its later amendments.

### Tissue capture and DNA extraction

In the experiments with next-generation sequencing, we randomly selected seven patients who were habitual smokers among the *H. pylori*-uninfected SRCCs.

In addition, two DNA samples (one from an SRCC within the mucosal layer and one from a deeper PDA lesion) were extracted from two cases with invasive SRCC without *H. pylori*-infected (Case 5 and 6).

We examined 10 diffuse-type gastric cancer associated genes (CDH1, TP53, ARID1A, KRAS, PIK3CA, ERBB3, FBXW7, TGFBR1, RHOA, and MAK2K1) referring to previous paper [10].

In the control group, seven samples were collected from only mucosal SRCC with *H. pylori*-infected. Therefore, in total, nine and seven DNA samples were extracted from the *H. pylori*-uninfected and -infected patients, respectively.

Pathologic tumor tissues were dissected from ten 10-μm-thick slides made from formalin-fixed paraffin embedded (FFPE) specimens, which were deparaffinized, stained, and dehydrated using the Arcturus® Paradise® PLUS Reagent System (Thermo Fisher Scientific, Waltham, MA, USA). Dissections were performed using a laser capture microdissection system (LMD 6500, Leica, Wetzlar, Germany), in accordance with the pathogenesis diagnosis. The DNA was extracted from these tissues using the GeneRead DNA FFPE kit (Qiagen, Valencia, CA, USA), and concentrations were determined using a Qubit® 1.0 fluorometer (Life Technologies, Carlsbad, CA, NY, USA). The quantity and quality of the FFPE-derived DNA samples were checked by calculating the normalized DNA integrity scores (ΔΔCq) via quantitative polymerase chain reaction (PCR) analysis using the Agilent NGS FFPE QC kit (Agilent, Santa Clara, CA, USA).

### Target enrichment and next-generation sequencing

DNA extracted from tumors was fragmented into 150–200 bp portions by sonication using an S2 sonicator (6 min, 10% duty, intensity = 5, 200 cycles/burst; Covaris, Woburn, MA, USA) and used for library construction according to the manufacturer’s instructions. In all cases, 10 ng of DNA was prepared for sequencing. The exons of ten oncogenes (*CDH1, TP53, ARID1A, KRAS, PIK3CA, ERBB3, FBXW7, TGFBR1, RHOA, and MAK2K1*) were enriched using the SureSelectXT HS Custom panel (Agilent). The resulting pooled libraries were quality control-checked via the High Sensitivity D1000 ScreenTape system using the 2200 TapeStation instrument (Agilent). Sequencing was performed with paired-end reads via the HiSeq 2500 platform (Illumina, San Diego, CA, USA).

### Variant detection

Sequencing reads were aligned to the hg19/GRCh37 reference sequence and analyzed using SureCall 4.0.1 (Agilent). PCR duplicates were removed and low-frequency mutations in template DNA molecules were detected using the molecular barcode system [11]. To identify variants in tumor samples, single sample analysis in SureCall 4.0.1 was used. The called variants were considered germline mutations if they were found in the dbSNP 137 or TogoVar (https://togovar.biosciencedbc.jp) databases. The remaining mutations in cancerous tissues were considered to be candidate cancer-specific mutations. To reduce the false positive rate, we set the cutoff values for somatic mutation in cancerous tissues as follows: variant score > 0.3; minimum quality for base > 30; variant call quality > 100; allele frequency > 0.1; and number of reads supporting the variant allele > 3 (Supporting file 1).

### Statistical analysis

All clinicopathological features were analyzed using the Chi-square test or Fisher’s exact test to compare categorical data and Student’s *t* test or Wilcoxon rank-sum test to compare continuous data. A *p-*value of < 0.05 was considered significant. All statistical analyses were performed using JMP® software (SAS International, Cary, NC. USA).

## Results

### Clinical features in *H. pylori*-uninfected and -infected SRCCs

First, we compared the clinicopathological features between 17 *H. pylori*-uninfected and -infected patients with SRCCs located within the mucosal layer. As shown in Table 1, the *H. pylori*-uninfected patients with mucosal SRCCs revealed male dominancy and a slightly younger age than the *H. pylori*-infected patients. The SRCCs in the *H. pylori*-uninfected patients tended to be located in the lower third of the stomach and its tumor size were significantly smaller than those in the *H. pylori*-infected patients. Although no difference was detected in blood type, family history of gastric cancer, or alcohol consumption.

**Table 1:** Comparison of *Helicobacter pylori*-uninfected and -infected mucosal signet ring cell carcinoma

	HpU-SRCC (*n* = 17)	HpI-SRCC (*n* = 34)	*p*-value
male/female	15/2	14/20	0.004
age	53.2 ± 11.4	58.2 ± 13.2	n.s.
location			
upper third	2 (11.8%)	0	n.s.
middle third	4 (23.5%)	24 (70.6%)	
lower third	11 (64.7%)	10 (29.4%)	
size	9.1 ± 5.3	16.0 ± 11.7	0.03
blood type (A/B/O/AB)	8/1/5/3	16/4/9/5	n.s.
family history^a^ of gastric cancer	6/13 (46%)	7/23 (30%)	n.s.
alcohol	10/17 (59%)	16/26 (62%)	n.s.
smoking	11/17 (65%)	6/26 (23%)	0.02
Brinkman Index	563.6	526.7	

HpU-SRCC: *Helicobacter pylori* (*H. pylori*)-uninfected signet-ring cell carcinoma, HpI-SRCC: *H. pylori*-infected signet-ring cell carcinoma, n.s.: not significant

^a^ Family History: relative within the 3-degree relationship

With drinking history, drink alcohol 21.6 g/day or more and drink more than 3 days a week as a drinker. And with smoking history, defined including smoking in present and in the past

### Genomic characteristics of SRCCs

DNA analysis by next-generation sequencing was performed in 16 samples from 14 patients (seven patients from each group) as shown in Table 2.

**Table 2:** Clinical characteristic of signet ring cell carcinoma subjected to gene analysis

No.	*H. pylori* infection	Age	Sex	Position	Size (mm)	Macroscopic type	Histology	Depth	Period^a^
1	negative	40–50	1	antrum	8	0-IIc	Sig	M	8y
2	negative	60–70	1	angular region	5	0-IIc	Sig	M	7y
3	negative	40–50	1	antrum	20	0-IIc	Sig	M	6y
4	negative	40–50	1	antrum	5	0-IIc	Sig	M	3y
5	negative	60–70	1	upper body	20	SMT	Por2 > Sig	SS	3y
6	negative	30–40	1	fornix	30	0-IIc	Por1 > Sig	SE	17y
7	negative	40–50	1	antrum	10	0-IIc	Sig	M	3y
8	positive	50–60	2	middle body	5	0-IIc	Sig	M	7y
9	positive	50–60	2	angular region	15	0-IIc	Sig	M	8y
10	positive	50–60	2	angular region	20	0-IIc	Sig	M	8y
11	positive	50–60	2	antrum	10	0-IIc	Sig	M	7y
12	positive	40–50	1	angular region	8	0-IIc	Sig	M	7y
13	positive	40–50	1	antrum	10	0-IIc	Sig	M	7y
14	positive	60–70	1	angular region	25	0-IIc	Sig	M	7y

*Sig* signet ring cell carcinoma, *por1* Poorly differentiated adenocarcinoma solid type, *por2* Poorly differentiated adenocarcinoma non-solid type, *M* mucosa, *SS* subserosa, *SE* serosa

^a^The period among paraffin embedding (years)

**Fig. 1:**
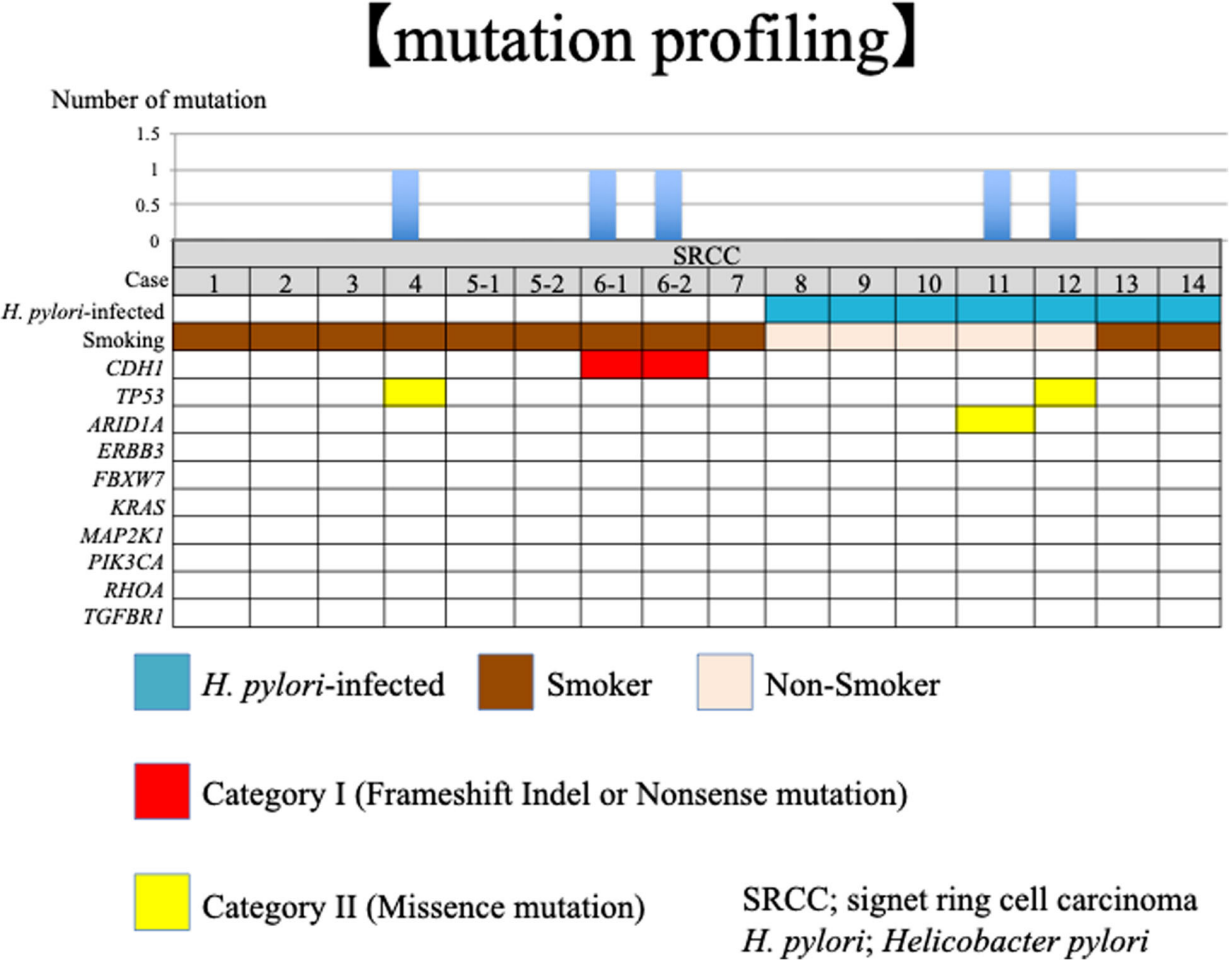
Mutational landscapes of Seven SRCCs without *H. pylori-*infected, and seven *H. pylori*-infected SRCCs. Gastric signet ring cell carcinoma (SRCCs) tissues, paired non-cancerous tissues, from Seven SRCCs without *H. pylori-*infected, and seven *H. pylori*-infected SRCCs were subjected to the targeted panel for 10 genes. The Upper bar-graph shows the number of somatic mutations per sample. The Lower panel shows the type of mutation of each gene. Genes reported SRCC-associated genes in previous paper. First line shows *H. pylori*-infected, second line shows smoking status, and from 3th to 12th line shows the type of mutation of each gene. Blue panels shows *H. pylori*-infected cases, brown panels shows present or current smoker, light-brown panels shows non-smoker, red panels shows frameshift indels or nonsense mutations, and yellow panels shows missence mutations

**Fig. 2:**
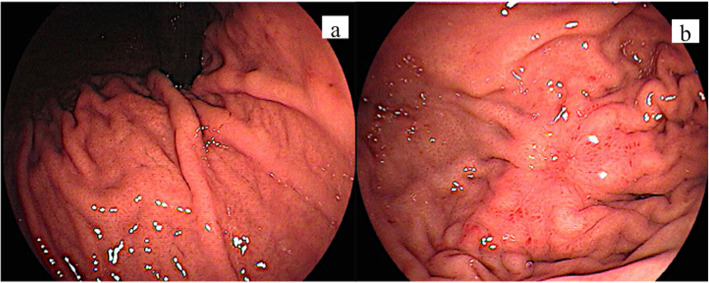
Endoscopic features of case 6 (a 30–40-year-sex 1). Endoscopic examination showed no atrophic changes in the background mucosa [**a**]. A depressed lesion measuring approximately 30 mm in diameter was observed in the fornix [**b**]

**Fig. 3:**
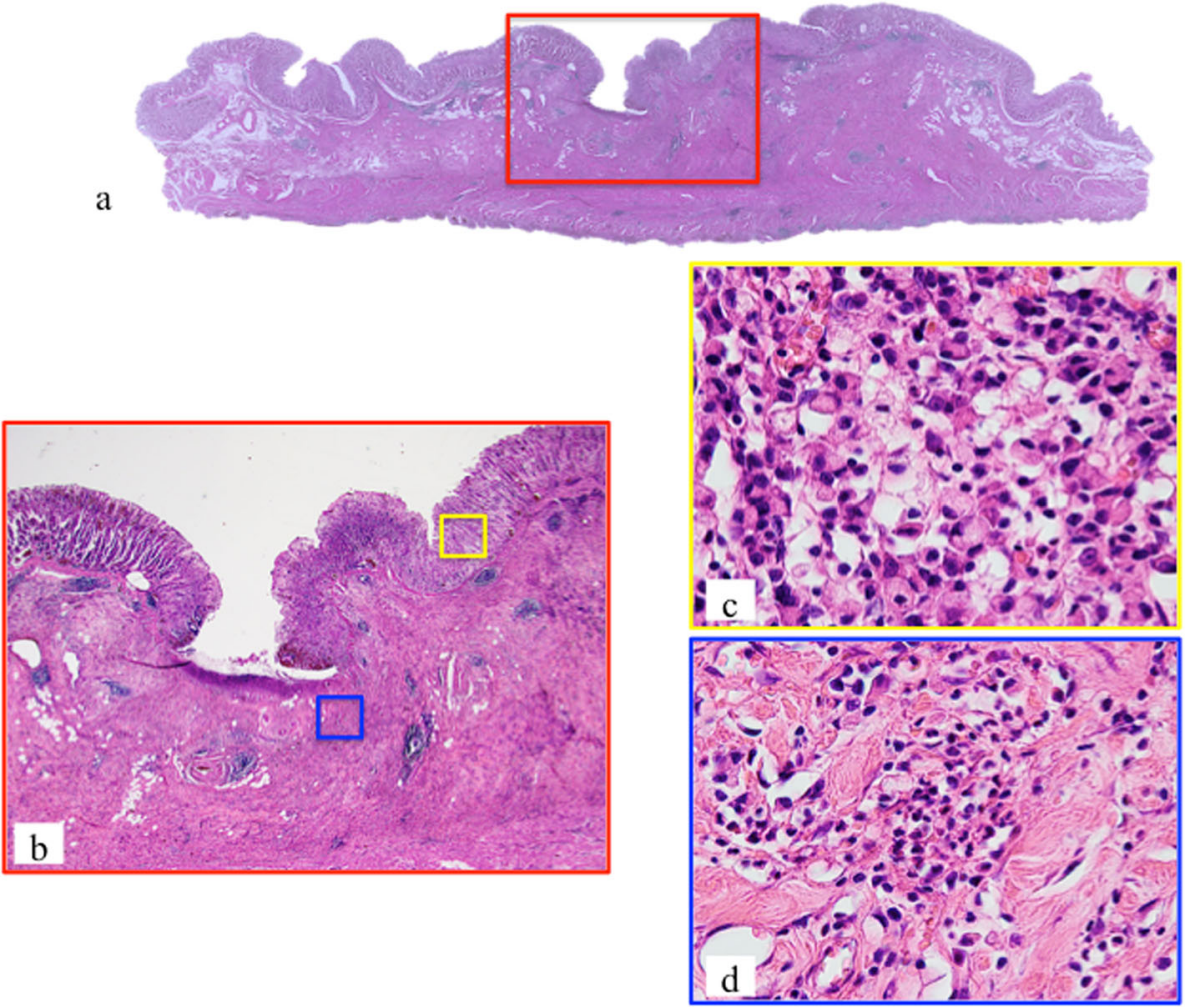
Histological features of case 6. A depressed lesion was found in the center of the section (a) and the low magnitude feature [red box in (**a**)] was demonstrated in (**b**). The histologic features of a higher magnitude mucosal signet ring cell carcinoma (yellow box) and a poorly differentiated adenocarcinoma in the deep invasive portion (blue box) are shown in (**c**) and (**d**), respectively (hematoxylin and eosin staining)

*A* single gene mutation was detected in each of five samples (Table 3, Fig. 1). A *CDH1* mutation (frame shift indel) was detected only in one *H. pylori*-uninfected cancer lesion (case 6) (Fig. 1). This mutation was found not only in the mucosal SRCC but also in the invasive PDA of that patient (Figs. 2 and 3). A *TP53* mutation was detected in one SRCC without *H. pylori*-infected (case 4). In the *H. pylori*-infected control group, mutations in *ARID1A* (case 11) and *TP53* (case 12) were detected in SRCCs (Fig. 1). No mutation in *CDH1* was found in the control group. On the other hand, we did not find somatic mutation in 7 genes (*KRAS, PIK3CA, ERBB3, FBXW7, TGFBR1, RHOA, and MAK2K1*) which associated somatic genomic alterations associated with the unique characteristics of sporadic diffuse gastric cancers [10]. (Fig. 1).

**Table 3:** Gene mutation detected in 16 samples

No.	Histology	Impact Gene	Chrom	Position	Ref Allele	Alt Allele	Allele Frequency	Read depth	Function Class	Codon	AA
4	Sig	TP53	17	7,579,542	C	G	0.39	251	MISSENSE	Gat/Cat	D10H
6–1	Sig	CDH1	16	68,853,327	TG	T	0.51	49			
6–2	Por1	CDH1	16	68,853,327	TG	T	0.57	69			
11	Sig	ARID1A	1	27,106,333	G	A	0.571	105	MISSENSE	Gtc/Act	V1765I
12	Sig	TP53	17	7,579,705	C	T	0.48	123	MISSENSE	Gtt/Att	V31I

*Sig* signet ring cell carcinoma, *por1* Poorly differentiated adenocarcinoma solid type

### The association between smoking status and, clinical and genomic characterization in SRCCs

As a result of comparison for smoking status in *H. pylori*-uninfected and -infected patients with SRCCs, the prevalence of smokers was significantly higher among the pylori-uninfected patients (*p* = 0.02). Although, the Brinkman index was not different between the two groups. Moreover, the C to A mutation, which is a characteristic smoking-induced mutation, was not found in any of the 17 *H. pylori*-uninfected mucosal SRCC.

## Discussion

In the present study, we examined the clinical and genetic characteristics of SRCCs in patients without *H. pylori*-infected. SRCCs in patients without *H. pylori*-infected showed different features compared with those in patients with *H. pylori*-infected. Clinically, the *H. pylori*-uninfected patients with mucosal SRCCs showed male dominance and the tumor tissue tended to be located in the lower third of the stomach. These observations imply that *H. pylori*-uninfected SRCCs have distinct biological characteristics and probably have a different carcinogenetic pathway as compared with *H. pylori*-infected SRCCs.

Particular focus should be placed on the genetic alterations detected in SRCCs without *H. pylori-*infected. Generally, diffuse-type cancers including SRCC are classified into the “genomically stable group” according to the four-subtype classification [12]. Under these conditions, *CDH1* have frequently been detected in SRCCs in patients with HDGC [13]. Moreover, E-cadherin, which is coded by *CDH1* gene, regulates signaling pathways leads to increase in cell proliferation, decrease in cell apoptosis followed by gastric cancer development [14]. However, in our study, the prevalence of mutations in *CDH1* was extremely low in SRCCs without *H. pylori-* infected. This suggests that the carcinogenic pathway of SRCCs without *H. pylori-* infected differs from that of SRCCs in patients with HDGC. The SRCC lesions found in *H. pylori*-uninfected patients seem to reflect not only a defect in cell-cell adhesion but also other neoplastic alterations.

Mutations in *TP53*, which are known as a representative driver mutation in gastric cancer carcinogenesis, were detected in SRCCs without *H. pylori-*infected as well as those with *H. pylori-*infected. This also strongly suggests that SRCCs in *H. pylori*-uninfected patients may be true neoplastic lesions. Previous reports have demonstrated that SRCC tissue gains an invasive ability after the mutation of *TP53* followed by conversion to PDA [15]. Accordingly, at least in a part of *H. pylori*-uninfected SRCC is a true neoplasm, and clinical features of SRCC without *H. pylori-*infected may be different from that in HDGC. In the present study, we demonstrated the presence of similar genetic alterations between mucosal SRCC and invasive PDA in a *H. pylori*-uninfected patient, suggesting the progression of SRCC to invasive PDA. Indeed, we have diagnosed two cases with invasive SRCC without *H. pylori-*infected (paper under submission). SRCC without *H. pylori-*infected may have a malignant potential to invade into the submucosal layer and should be treated as a diffuse-type gastric cancer as described in the guidelines for treatment [16].

Notably, it should be emphasized that the prevalence of smokers was higher among the *H. pylori*-uninfected patients with SRCCs than among the *H. pylori*-infected patients. This finding is compatible with the previous report by Horiuchi et al. [8]. However, in our study concerning the genetic alterations in SRCC tissue samples, we could not find any C to A mutations, which are recognized as smoking-induced alterations, despite including samples from some heavy smokers in our analysis [17, 18]. Smoking may be a confounding factor as to the pathogenesis of SRCCs without *H. pylori*-infected.

In addition to genetic mutations, gastric carcinogenesis may also be influenced by epigenomic alterations including gene methylation. Epstein–Barr virus (EB virus) infection, which induces several epigenetic alterations in host genes, may be another carcinogenetic factor besides *H. pylori-*infected [19]. However, it is supposed that *H. pylori-*infected is essential for EB virus-induced carcinogenesis [20]. The pathogenesis of SRCCs without *H. pylori-*infected remains unclear and should be clarified in future research.

This study has several limitations. The first limitation is the limited number of patients. To accurately evaluate the prevalence of any given genetic mutation, a large number of cases should be examined. In addition, we examined only ten genes in our cancer panel and we could not cover their complete exon sequences. In clinical practice, SRCCs without *H. pylori-*infected are diagnosed when the lesions measure about 10 mm in diameter; therefore, it is difficult to extract high quality DNA from SRCC tissue. The second limitation is that DNA samples from control lymphocytes were not analyzed. Therefore, we excluded disease-specific germline mutations and Japanese healthy germline mutations when searching the dbSNP 137 and TogoVar databases.

## Conclusions

Our findings indicate that SRCCs without *H. pylori* should be treated as having a malignant potential similar to that for SRCCs occurring in the presence of *H. pylori*-infected*.* Smoking may not be the main carcinogenic factor for the development of SRCCs among the *H. pylori*-uninfected patients.

## Supplementary information

**Additional file 1.**

## Data Availability

The datasets used and analyzed during the current study will be available from the corresponding author on reasonable request.

## Abbreviations

SRCC: Signet ring cell carcinoma
*H. pylori*: *Helicobacter pylori*
HDGC: Hereditary diffuse gastric cancer
PDA: Poorly differentiated adenocarcinoma
FFPE: Formalin-fixed paraffin embedded
EB virus: Epstein–Barr virus
