# Supplementary material for: Clinical and genomic characteristics of mucosal signet-ring cell carcinoma in Helicobacter pylori-uninfected stomach
Source: BMC Gastroenterol. 2020 Jul 29;20:243. doi: 10.1186/s12876-020-01387-9 (PMC7391816; doi:10.1186/s12876-020-01387-9)
Supplement: Supplementary file 1 — Additional file 1. [file 12876_2020_1387_MOESM1_ESM.docx]

**Variant score**; This value is the minimum quality score that a mutation call must have in order to be included in the results. Calls with a quality score below this value are excluded from the results. 0.3 means that the accuracy of the call is 99%.

**Minimum quality for base;** This value is the minimum quality score that a base must have in order to be called as a mutation within a read. The value helps ensure that low-quality bases are not falsely called as mutations. Lowering the value could cause an increase in the number of mutation calls, including false positive calls. Conversely, raising the value could lead to fewer mutation calls.

**Variant call quality**; This value is the minimum Phred quality score that a candidate low frequency mutation needs to have in order to be reported in the results.

**Allele Frequency;** This value is the minimum allele frequency that a potential mutation call must have in order for the program to call it as a mutation. Potential mutation calls with an allele frequency below this threshold are not called as mutations.

**Number of reads supporting variant allele;** This parameter sets the minimum number of reads that support the variant allele sequence and also pass the quality filters. The default of 3 means that the sequencing data for the sample must have at least 3 reads that pass the quality filters and supports the variant allele sequence. The number of reads specified in this parameter refers to merged reads of a consensus sequence. Each merged read is the result of a set of one or more raw reads that was ultimately merged into a single consensus sequence.
